# Validation of a new method for immobilising kinetoplastid parasites for live cell imaging

**DOI:** 10.1016/j.molbiopara.2009.09.008

**Published:** 2010-01

**Authors:** Helen P. Price, Lorna MacLean, Joanne Marrison, Peter J. O’Toole, Deborah F. Smith

**Affiliations:** a Centre for Immunology and Infection, Department of Biology/Hull York Medical School, University of York, Heslington, York YO10 5YW, UK; b Technology Facility, Department of Biology, University of York, Heslington, York YO10 5YW, UK

**Keywords:** BSF, bloodstream form, FRAP, fluorescence recovery after photobleaching, GFP, green fluorescent protein, PCF, procyclic form, ROI, region of interest, *Trypanosoma brucei*, *Leishmania major*, Live cell imaging, FRAP

## Abstract

The kinetoplastid parasites are responsible for three of the ten most neglected tropical diseases as classified by the WHO. Recent advances in molecular and cellular analyses have allowed rapid progress in our understanding of the biology of these lethal pathogens. In this study we validate a new method for immobilising *Trypanosoma brucei* and *Leishmania major* parasites while maintaining a high level of viability. This allows reproducible live cell imaging of these highly motile organisms, thus enabling a full complement of advanced microscopic techniques to be utilised to better understand these pathogenic species.

## Graphical abstract

Here we validate a new method for immobilising the protozoan parasites *Trypanosoma brucei* and *Leishmania major* parasites while maintaining a high level of viability.

Neglected tropical diseases affect over 1 billion people in some of the poorest and most unstable regions of the world. Three of the most severe of these infections are caused by kinetoplastid parasites, *Leishmania spp*., *Trypanosoma brucei* and *T. cruzi*, for which treatment depends to a greater or lesser extent on drugs with unacceptable levels of toxicity. There is an urgent need for research leading to novel therapeutics to control these infections. In addition to its clinical relevance, *T. brucei* has also emerged as a powerful model organism for research on eukaryotic cell biology, including mechanisms of intracellular trafficking and organelle biogenesis [1–3].

Live cell imaging is an invaluable tool in the study of eukaryotic cellular function, allowing real-time capture of fundamental processes at the individual cell level. Analysis of live cells by advanced microscopy techniques such as FRAP and FRET can also provide critical insights into molecular diffusion and protein complexing [4]. In order to produce accurate data, there must be an effective and reproducible method in place for the total immobilisation of cells in a state of optimal health. Technical difficulties with cell viability are compounded in the case of the flagellated parasitic protozoa by rapid motility, which is essential for viability at least in *T. brucei* [2]. Previous studies have used agarose to mount live parasites [3] but this method is technically challenging, does not consistently produce total immobilisation and the effects of agarose on parasite viability have not been studied in detail.

Here we describe validation of a rapid method for immobilising live *Leishmania major* and *T. brucei* using the new formulation of a thermoreversible gel CyGEL (Biostatus Ltd., UK). This is an optically clear compound which is liquid when ice-cold but forms a solid matrix upon warming to 15 °C and above. The gel can also act as a controlled delivery system for a range of fluorescent probes, including FM4-64 and propidium iodide.

We tested the effects of CyGEL on the viability of three cell types: *L. major* procyclic promastigotes (vector-transmitted extracellular stages), *T. brucei* procyclic (PCF, vector-transmitted extracellular stages) and *T. brucei* bloodstream form (BSF, extracellular parasites resident in the host). First, we incubated cells in microcentrifuge tubes in PBS-primed CyGEL at 20 °C for up to 3 h, before washing in ice-cold PBS and assessing viability by flow cytometry using the cell impermeant dye, propidium iodide (Fig. 1A) to facilitate analysis of large numbers of cells. Experimental conditions were initially well tolerated by both *L. major* and *T. brucei* insect stage parasites, with less than 5% decrease in cell viability observed following a 2 h incubation in the matrix (Fig. 1A). After 3 h, viability had decreased to approximately 80% for *L. major* but remained above 90% for *T. brucei* procyclic cells. Results for *T. brucei* BSF are discussed below.

We then compared the effects of cell immobilisation in either CyGEL or 3% agarose/PBS on glass slides, measuring viability by propidium iodide exclusion. Both *L. major* and *T. brucei* PCF tolerated CyGEL immobilisation on slides for a period of 3 h (Fig. 1B), although viability decreased at a faster rate in *T. brucei* PCF than in *L. major* promastigotes. No visible motility was seen over this period for either cell type. Swelling became apparent in both parasite species beyond the 3 h time point, perhaps as a result of incomplete cell division (data not shown). This change in morphology was followed by a considerable decrease in cell viability in both cell types by 4–5 h post-immobilisation (Fig. 1B). Technical issues prevented the collection of accurate data for cells immobilised in agarose, which proved to be a poor delivery system for propidium iodide. Staining was patchy and all slides contained several regions where the cells were either highly motile or were dead due to dehydration.

Longer term effects of CyGEL immobilisation were studied by recovering treated cells by cooling and washing in ice-cold PBS, then placing them back into *in vitro* culture. The *L. major* promastigotes were most resistant to long term damage and successfully re-established in culture even after incubation in the gel for 3 h (Fig. 1C). Some irreversible damage was seen in *T. brucei* PCF, as cells incubated in the matrix for 2 h or longer showed restricted motility on recovery (data not shown) and defective growth in culture (Fig. 1D). There was no comparable method available for the full recovery of all agarose-immobilised parasites.

In contrast to procyclic cells, mammalian host stage BSF *T. brucei* were much more sensitive to immobilisation by CyGEL and showed approximately 30% cell death after 15 min in microcentrifuge tubes (data not shown) and 90% death after 1 h (Fig. 1A). We then analysed the effects of mounting BSF on glass slides in three different compounds: PBS-primed CyGEL (as used above), PBS-primed CyGEL containing 10 mM glucose and CyGEL-Sustain (with RPMI), which is recommended by the manufacturer for longer incubation times. Immobilisation in CyGEL (+/− glucose) caused clumping, swelling and a rapid decrease in cell viability (Fig. 1E, F and data not shown). In correlation with these data, recovered cells were unable to re-establish in *in vitro* culture following incubation in the matrix for 30 min (data not shown). Treatment with CyGEL-Sustain also caused rapid swelling (Fig. 1F) but, unlike in CyGEL, complete immobilisation was not achieved and viability decreased at a much slower rate (Fig. 1E). While CyGEL compounds do appear to be toxic to BSF cells, it is also interesting to note that genetic studies have shown motility to be critical for the viability of the host bloodstream form but not the procyclic form of *T. brucei* [2], although the kinetics of this essentiality have not been determined.

We conclude from these data that CyGEL is highly suitable for imaging *L. major* promastigotes and *T. brucei* PCF for up to 2 h and is particularly appropriate for use when complete immobilisation is important (e.g. FRAP analysis, see below). Slide preparation time was much shorter for CyGEL than agarose and results were considerably more reproducible. The morphology of cells immobilised in CyGEL does deteriorate after an extended time; therefore we would recommend the use of agarose for experiments requiring imaging for more than 2 h, especially in situations where some restricted movement can be tolerated. In contrast to our findings for insect stage parasites, immobilisation of *T. brucei* BSF in CyGEL (+/− glucose) caused rapid cell death and so is not appropriate for use on this cell type. Our investigations also revealed that a related formulation, CyGEL-Sustain, did not produce complete immobilisation of any of the three cell types tested and so is not suitable for these species using current methods.

Following these viability assays, we proceeded to study the possible applications of CyGEL for the study of insect stage kinetoplastids. We successfully employed this immobilisation method to perform FRAP analysis on GFP-expressing *L. major* promastigotes (Fig. 2A and B as a movie in Supplementary data 1). FRAP experiments demonstrated the ability to accurately record the diffusion rates of GFP within live *L. major* promastigotes. As expected the freely diffusing cytosolic GFP showed rapid recovery (*t*½ ≤ 1 s) within the bleached region of interest (ROI). The recovery was less than 100% due to a significant proportion of GFP bleaching during the bleach pulse itself. The bulk of the surrounding area equilibrated to the same intensity as the bleached ROI, demonstrating that there was no membrane-bound fraction.

It was also possible to use the CyGEL matrix as a delivery system for subcellular markers. The uptake of the lipophilic dye FM4-64 was monitored in real time in the insect form stages of both *L. major* (as a time-lapse movie in Supplementary data 2) and *T. brucei* (Fig. 2C). This dye initially binds to the plasma membrane and is taken up at the flagellar pocket (the site of all endo- and exocytosis in these organisms), before trafficking through the endocytic pathway, terminating in the single lysosomal compartment (Fig. 2C). Images were collected at various time points while the organisms were held in CyGel over a 90 min time frame. CyGEL also proved to be a rapid and efficient delivery system for Lysotracker which accumulates in the lysosome, late endosomes and acidocalcisomes, and the mitochondrial-specific probe Mitotracker. These probes were used to stain live *L. major* promastigotes expressing the first 18 amino acids of the dually acylated protein HASPB fused to a C-terminal GFP tag, which localises to the plasma membrane and flagellar pocket [5] (Fig. 2D and E).

In conclusion, we have validated the thermoreversible gel CyGEL as a suitable mounting medium for the immobilisation and live cell imaging of the insect stages of two protozoan parasites, *L. major* and *T. brucei*, with less than 20% drop in cell viability over 2 h. The methods we describe here will have a wide range of applications within the field of parasite cell biology.

## Figures and Tables

**Fig. 1:**
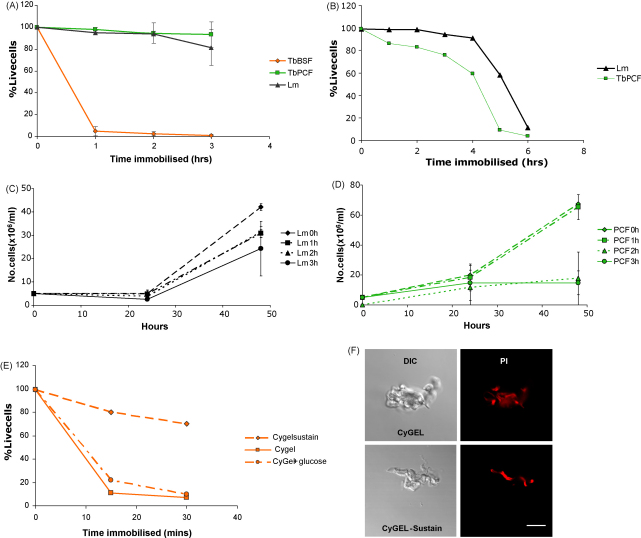
Parasite viability following CyGEL treatment. (A) Cell viability was determined after immobilisation in CyGEL in tubes. Briefly, logarithmically growing parasites (1 × 10^7^*L. major* promastigotes and *T. brucei* PCF, or 1 × 10^6^*T. brucei* BSF) were washed in PBS and resuspended in <10 μl PBS in a microcentrifuge tube before the addition of 200 μl of ice-cold PBS-primed CyGEL (Biostatus Ltd., UK). Samples were incubated at RT for 0–3 h, then placed on ice to allow the matrix to liquify. Cells were washed with ice-cold PBS and stained in 5 μg/ml propidium iodide/PBS. FACS analysis of >10,000 cells per sample was performed using a Cyan ADP analyser. Tb BSF, *T. brucei* bloodstream form strain Lister 427; Tb PCF, *T. brucei* procyclic form strain 449; Lm, *L. major* procyclic promastigotes strain MHOM/IL/81/Friedlin. (B) Cell viability was determined following immobilisation in CyGEL on glass slides. *L. major* promastigotes and *T. brucei* PCF (as above) were washed in PBS and resuspended in <10 μl PBS before the addition of 200 μl of ice-cold PBS-primed CyGEL containing 5 μg/ml propidium iodide. Each suspension was mixed briefly by flicking the tube, then aliquotted onto 3 glass coverslips (22 mm × 40 mm) on several layers of tissue paper. A glass slide was added before briefly transferring to an ice pack to allow the mixture to spread out. Samples were then incubated at 20 °C for 5 min, sealed with nail varnish and imaged by confocal microscopy. Propidium iodide exclusion was used as the marker of viability. >200 cells were counted per sample for each time point. Growth of *L. major* promastigotes (C) and *T. brucei* PCF (D) *in vitro* following immobilisation in CyGEL. Cells were immobilised for 0–3 h as described in A, washed in ice-cold PBS, then placed in appropriate culture medium and incubated at 26 °C for 48 h, counting on a haemocytometer at 24 h intervals. Data were collected from at least three independent experiments. (E) Viability of *T. brucei* BSF immobilised on glass slides. 1 × 10^6^ cells were washed in PBS and mounted as described in B, in PBS-primed CyGEL+/− 10 mM glucose or in CyGEL-Sustain (prepared with 10× RPMI according to the manufacturer's instructions), all containing 5 μg/ml propidium iodide. >200 cells were counted per sample for each time point. (F) Representative images of *T. brucei* BSF immobilised in CyGEL or CyGEL-Sustain for 15 min. PI, propidium iodide. Bar, 10 μm.

**Fig. 2:**
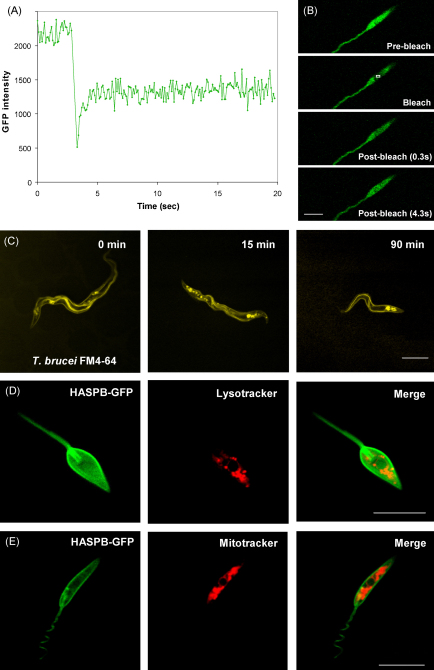
Live cell imaging of parasites immobilised in CyGEL. (A and B) FRAP analysis. *L. major* promastigotes were transfected with the construct pSSu-int/eGFP [6] using methods described previously [7] to produce a stable transgenic line constitutively expressing eGFP. Cells were immobilised in PBS-primed CyGEL (as above) and visualised using a Zeiss LSM 510 meta confocal microscope with a Plan-Apochromat 63×/1.4 Oil DIC I objective. GFP was excited using the 488 nm laser and emission collected through a 505 LP filter. Regions of interest (ROI) were chosen for FRAP experiments. The laser was then scanned only in the selected ROI with 100 iterations at an elevated laser power. Pre- and post-images were collected as part of a time series. Analysis was performed using SigmaPlot 11 and fit according to a single exponential. FRAP analysis on this cell line showed rapid recovery (*t*½ ≤ 1 s) indicative of free cytosolic GFP. The bleached region of interest is shown as a white box in the second panel of B. Bar, 5 μm. (C) Trafficking of the lipophilic dye FM4-64 in *T. brucei* PCF. Cells were washed in PBS and treated with 40 μM FM4-64 (Invitrogen) prior to CyGEL immobilisation using described methods [8]. Samples were visualised by confocal microscopy, with FM4-64 excited at 543 nm and emission collected through a 560 LP filter. The dye was seen on the plasma membrane and at the flagellar pocket at time 0. By 15 min the signal appeared in the endosomal system, reaching the terminal lysosome by 90 min. Efficient staining was also achieved when cells were immobilised in CyGEL containing 40 μM FM4-64 (see time-lapse movie in Supplementary data 2) although the initial uptake of dye was slightly delayed using this method. (D and E) Images of *L. major* promastigotes expressing the first 18 amino acids of the dually acylated protein HASPB fused to a GFP tag, which localises to the plasma membrane and flagellar pocket [5]. 1 × 10^7^ cells were washed in PBS and resuspended in <10 μl PBS in a microcentrifuge tube before the addition of 200 μl of ice-cold PBS-primed CyGEL containing 50 nM of either Lysotracker Red DND-99 (D) (Invitrogen) or Mitotracker Deep Red 633 (E) (Invitrogen). Samples were visualised by confocal microscopy. GFP, Lysotracker and Mitotracker were excited using the 488 nm, 543 nm and 633 lasers and emission collected through 505, 560 and 650 LP filters, respectively. Lysotracker probes accumulate in low pH compartments, therefore targeting the late endosomes, lysosome and acidocalcisomes in *L. major*. Mitotracker probes have a mildly reactive chloromethyl moiety which label active mitochondria, dependent on membrane potential. The images shown here are taken from cells immobilised for 30 min (E) or 2 h (D). The cell shown in D has initiated cell division, as evident by the presence of two flagellar pockets. Bar, 10 μm.

## Appendix A Supplementary data

Supplementary data 1 Time-lapse movie showing FRAP analysis on a CyGEL-immobilised *Leishmania major* promastigote expressing GFP, as shown in Fig. 2A and B. There was rapid recovery (*t*½ ≤ 1 s) following photobleaching, which is indicative of free cytosolic GFP.

Supplementary data 2 Time-lapse movie showing FM4-64 trafficking through a *Leishmania major* promastigote following immobilisation in PBS-primed CyGEL containing 40 μM FM4-64. Confocal microscopy was performed as described for Fig. 2C, collecting images every 2 min for 90 min (of which the first 30 min are shown here). The dye is initially found at the plasma membrane and flagellar pocket, is taken up and transported through the endocytic system, accumulating in the terminal endocytic compartment by 30 min.
