# Supplementary figures and images for: Validation of a new method for immobilising kinetoplastid parasites for live cell imaging
Source: Mol Biochem Parasitol. 2010 Jan;169(1-10):66–9. doi: 10.1016/j.molbiopara.2009.09.008 (PMC2791879; doi:10.1016/j.molbiopara.2009.09.008)

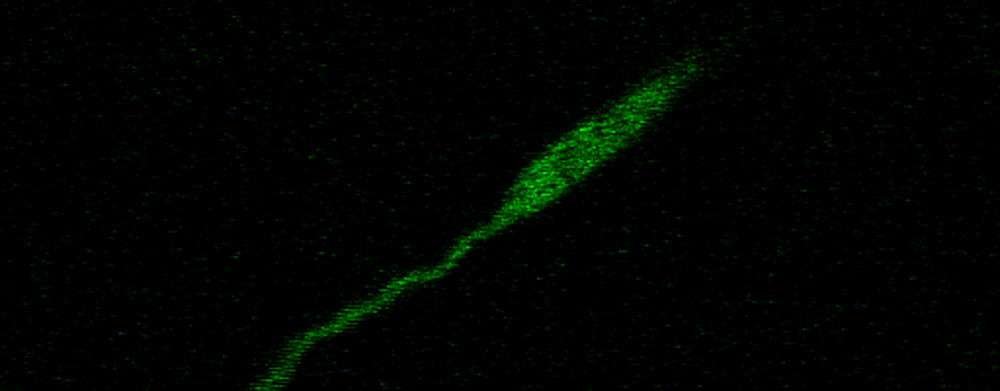

Supplement: Supplementary data 1 — Time-lapse movie showing FRAP analysis on a CyGEL-immobilised Leishmania major promastigote expressing GFP, as shown in Fig. 2A and B. There was rapid recovery (t½ ≤ 1 s) following photobleaching, which is indicative of free cytosolic GFP. [file mmc1.jpg]

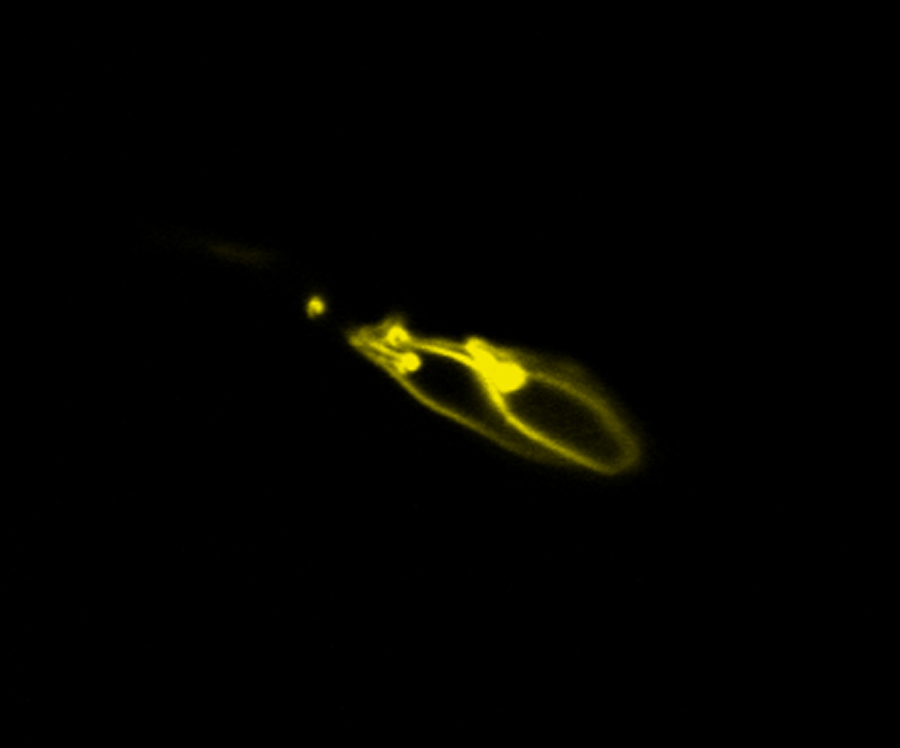

Supplement: Supplementary data 2 — Time-lapse movie showing FM4-64 trafficking through a Leishmania major promastigote following immobilisation in PBS-primed CyGEL containing 40 μM FM4-64. Confocal microscopy was performed as described for Fig. 2C, collecting images every 2 min for 90 min (of which the first 30 min are shown here). The dye is initially found at the plasma membrane and flagellar pocket, is taken up and transported through the endocytic system, accumulating in the terminal endocytic compartment by 30 min. [file mmc2.jpg]

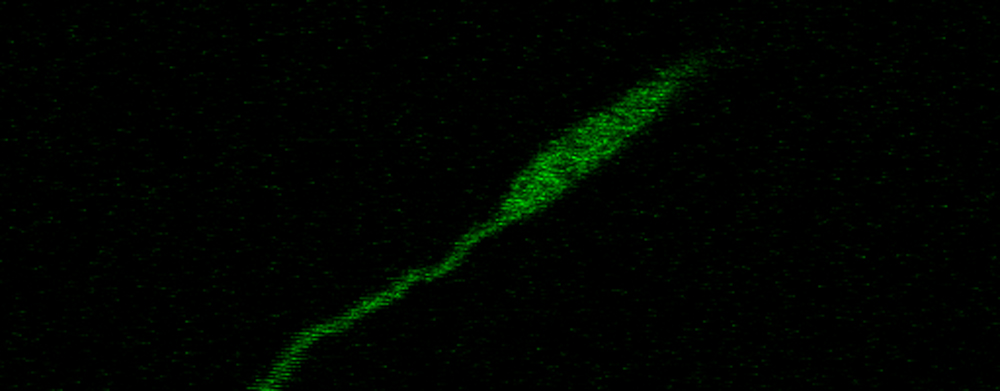

Supplement: Supplementary file 3 [file mmc3.jpg]

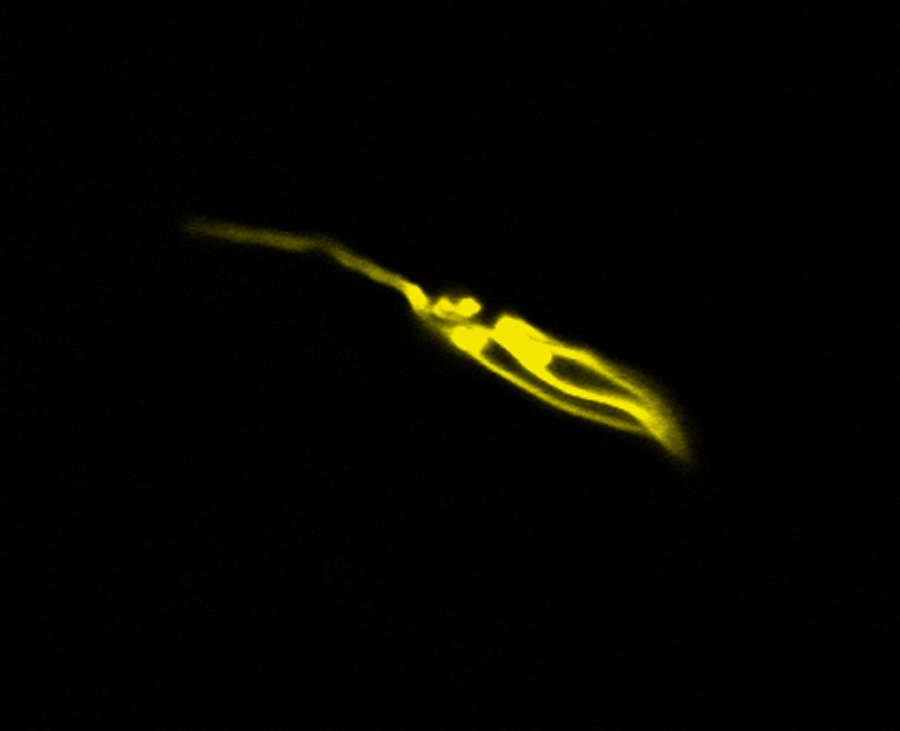

Supplement: Supplementary file 4 [file mmc4.jpg]
